# Methyl Paraben Affects Porcine Oocyte Maturation Through Mitochondrial Dysfunction

**DOI:** 10.3390/biom14111466

**Published:** 2024-11-18

**Authors:** Huimei Huang, Chuman Huang, Yinghua Li, Xingwei Liang, Namhyung Kim, Yongnan Xu

**Affiliations:** 1 Guangdong Provincial Key Laboratory of Large Animal Models for Biomedicine, South China Institute of Large Animal Models for Biomedicine, School of Pharmacy and Food Engineering, Wuyi University, Jiangmen 529000, China; 2112207086@wyu.edu.cn (H.H.); 18318843832@163.com (C.H.); yhli@wyu.edu.cn (Y.L.); 2 College of Animal Science & Technology, Guangxi University, Nanning 530004, China; xwliang@gxu.edu.cn

**Keywords:** methyl para-hydroxybenzoate, meiosis, redox imbalance, mitochondrial dysfunction

## Abstract

Parabens are widely used in various industries, which are including chemical, pharmaceutical, food, cosmetic, and plastic processing industries. Among these, methyl paraben (MP) serves as an antimicrobial preservative in processed foods, pharmaceuticals, and cosmetics, and it is particularly detected in baby care products. Studies indicate that MP functions as an endocrine-disrupting compound with estrogenic properties, negatively affecting mitochondrial bioenergetics and antioxidant activity in testicular germ cells. However, limited information exists regarding studies on the effects of MP in oocytes. The aim of this study was to investigate the specific mechanism and the toxic effects of MP during oocyte maturation cultured in vitro using a porcine oocyte model. The results indicated that MP (50 μM) inhibited oocyte expansion, significantly reducing the expression of expansion-related genes *MAPK1* and *ERK1*, and decreased the first polar body extrusion significantly as well. ATP levels decreased, reactive oxygen species (ROS) levels remained unchanged, and glutathione (GSH) levels decreased significantly, resulting in an elevated ROS/GSH ratio. The expression of antioxidant genes *SOD1* and *GPX* was significantly decreased. Additionally, a significant decrease in levels of mitochondrial production and biosynthesis protein PGC1α+β, whereas levels of antioxidant-related protein Nrf2 and related gene expression were significantly increased. Autophagy protein LC3B and gene expression significantly decreased, and apoptosis assay indicated a significant increase in levels of caspase3 protein and apoptosis-related genes. These results demonstrated the negative effect of MP on oocyte maturation. In conclusion, our findings indicate that MP disrupts redox balance and induces mitochondrial dysfunction during meiosis in porcine oocytes, resulting in the inhibition of meiotic progression. The present study reveals the mechanism underlying the effects of methyl para-hydroxybenzoate on oocyte maturation.

## 1. Introduction

Methyl paraben (MP) belongs to a homologous series of parabens known for their antimicrobial effects and is widely used as a preservative in food, pharmaceuticals, and cosmetics. MP has been detected in 60% of products, with infants and children ingesting three times as much through the skin as adult women do from products containing MP [1]. Andersen et al. reported that the toxic effects of MP can cross the placental barrier, implying potential fetal exposure [2]. Moreover, recent studies have demonstrated the risk of endocrine system disruption associated with the use of MP. These findings collectively indicate potential risks to the environment and human health [3] and suggest higher toxicity risks to embryos and infants.

Meanwhile, increasing evidence indicates the presence of MP in the atmosphere and their potential health risks to humans and other organisms. Recent studies have detected parabens in surface and tap water due to their use as disinfectants; birds of prey from Texas and North Carolina (USA) exhibited detectable levels of MP in over 50% of examined tissues, with a notably higher accumulation observed in liver samples [4]. Beyond water pollution, the atmospheric release of MP from domestic waste also represents a significant biological health threat. Nielsen NJ et al. [5] assessed the risk status associated with microplastic components for both human health and environmental safety following anaerobic treatment of domestic waste; gas chromatography mass spectrometry (GC-MS) analysis revealed that the peak height of detected MP was reduced by more than twofold or remained unaffected by anaerobic digestion, suggesting its persistence post-treatment. In addition to posing potential health threats to terrestrial organisms, Dasmahapatra AK et al. [6] demonstrated that the release of MP into aquatic ecosystems constitutes a significant risk for aquatic life forms, including fish. MP can act as an endocrine disruptors, impeding gamete development and growth while also inducing behavioral changes and neurological disorders in fish.

Molecular-level studies have reported that the mechanism of toxic action of MP may be related to mitochondrial failure. However, the inhibitory effect of MP on membrane transport and mitochondrial functional processes is generally considered significant [7]. In vivo administration experiments in mice and embryonic experiments in zebrafish have demonstrated the effect of MP on antioxidant capacity during reproductive development, exhibiting genotoxicity in embryos and reproductive organs [8,9]. However, the toxic effects of MP in the maturation of human oocytes and its mechanism remain unclear. Meanwhile, it is difficult to obtain immature human oocytes, and the sources are relatively few. In this study, pig oocytes are selected to replace them. Pigs and humans are mammals, anatomically and physiologically similar to humans. The life process of porcine oocytes is similar to that of human oocytes, and the small molecule mechanism of life activity is highly similar. Studies have shown that maternal mRNA is affected by deadenylation-dependent decay during oocyte maturation in mice, rats, pigs, and humans [10]. Therefore, in this study, porcine oocytes were used to study the mechanism of MP’s effect on oocytes, which can reasonably reflect the threat of MP to human reproductive health.

Oocyte maturation is a complex process involving the regulation of numerous small molecules. Upon luteinizing hormone (LH) surge, primordial follicles are stimulated to develop to the preovulatory follicular stage. Meiotic resumption in pre-MI is characterized by the disappearance of the nuclear membranes of oocytes (blastomeres), known as germinal vesicle breakdown (GVBD). Following GVBD and MI completion, the oocyte proceeds to meiosis II, where the mature oocyte extrudes half of its chromosomes into the polar body and advances to metaphase at mid-phase II (MII) until fertilization. Discharge of the first polar body (PBI) is the primary indicator of oocyte maturation [11,12]. Perivitelline cells of the oocyte are reported to control the meiotic progression of the oocytes, greatly influencing oocyte maturation and development [13]. The influence index of granulosa cells is primarily assessed by the degree of expansion of the oocyte. Additionally, the expression of oocyte expansion-dependent genes, such as *P53*, *ERK1*, *MAPK1*, and *TPX2*, is crucial in influencing oocyte maturation [14,15,16,17]. Determining the expression of expansion genes can contribute to assessing the mechanism influencing the maturation of oocytes.

The major function of mitochondria is to produce ATP for normal cellular life activities. Numerous studies have established the influence of mitochondria function on the normal developmental processes of germ cells. Amaral S et al. reported a close association between mitochondrial function and sperm homeostasis, underscoring the growing significance of understanding the impact of various compounds, from dietary supplements to environmental pollutants, on sperm mitochondria [18]. The vitality of mature oocytes and subsequent embryos is highly reliant on the functionality of oocyte mitochondria. In their review, Kelli F Malott et al. highlighted the adverse effects of toxic substances on mitochondria, emphasizing their susceptibility to mitochondrial damage. They emphasized the substantial demand for ATP during oocyte maturation, which is essential for continuous transcription and translation, underscoring the importance of obtaining an adequate quantity of functional mitochondria [19]. Kirillova A et al. further highlighted the importance of mitochondria for oocyte maturation. They demonstrated that in vitro matured oocytes undergo significant changes in mitochondrial distribution and alterations in the expression of proteins related to mitochondrial function as well as associated coding genes during maturation under external conditions [20]. Therefore, the assessment of mitochondrial distribution and function in oocytes matured in vitro in a medium supplemented with MP aids in elucidating the mechanisms of oocyte maturation.

Glutathione (GSH), as the most essential non-enzymatic antioxidant, holds immense significance in sustaining the meiotic process. Increased GSH content in oocytes plays a key role in protecting oocytes against oxidative stress-induced apoptosis [21]. Concurrently, the reactive oxygen species (ROS)/GSH balance influences cellular physiological functions and is essential for promoting oocyte maturation [22]. Enzymatic systems play a pivotal role in oocyte maturation by mitigating damage from oxidative stress to ensure optimal development. In vitro experiments have demonstrated that antioxidant drugs promote the nuclear translocation of nuclear factor erythroid 2-related factor 2 (Nrf2). This process transcriptionally upregulates the expression of superoxide dismutase and glutathione peroxidase, thereby eliminating excess ROS, preventing mitochondrial damage, and enhancing oocyte quality [23]. Toxic substances activate the Nrf2 pathway and impede oocyte maturation by inducing oxidative stress, resulting in diminished porcine oocyte quality, with melatonin demonstrating a reversal of this effect [24]. Nrf2 is crucial in counteracting oxidative stress during oocyte maturation.

The aim of this study was to investigate the effect of MP exposure on porcine oocyte maturation and its underlying mechanism. The release of oocyte polar bodies served as the primary indicator of maturation, accompanied by the assessment of the degree of oocyte mound expansion and the expression of maturation-related genes. Immunofluorescence staining and RT-qPCR were further used to validate the effect of MP exposure on oocyte maturation and elucidate the specific mechanism involved.

## 2. Research Methods

### 2.1. Antibodies and Chemicals

MP(HY-N0349) was purchased from MedChemExpress (Junction, NJ, USA). All chemicals and reagents, unless otherwise specified, were procured from Sigma-Aldrich (St. Louis, MO, USA).

### 2.2. Oocyte Maturation In Vitro and Analysis of Cumulus Expansion

Porcine ovaries used in the experiment were obtained from sows slaughtered in the morning at an adjacent abattoir. The obtained ovaries were placed in thermos flasks containing sterile saline at 37 °C. After transportation to the laboratory, the ovaries were washed three times with sterile saline. Using a sterile syringe (18-gauge needle), follicles 3–6 mm in diameter containing cumulus oocyte complexes (COCs) were collected. The COCs were washed five times with 4(2-hydroxyethyl)-1-piperazineëthanesulfonic acid (HEPES) before being transferred to fresh in vitro maturation (IVM) medium (M199 medium containing 0.022 mg/mL sodium pyruvate, 10% porcine follicular fluid, 0.09 mg/mL L-cysteine, 1% penicillin–streptomycin, 10 IU/mL follicle-stimulating hormone, 20 ng/mL epidermal growth factor, and 10 IU/mL LH). Four-well culture plates were filled with 500 µL IVM medium and approximately 50–60 oocytes per well, and each well was covered with 500 µL mineral oil to submerge the IVM medium and oocytes completely. Oocytes were matured in vitro at 38.5 °C (5% CO_2_, 100% humidity) for 44–46 h. During this period, the IVM medium did not need to be replaced.

To evaluate the extent of cumulus expansion, groups of COCs will be cultured for 46 h, which means oocytes have no treated and treated with different concentrations (5, 50, and 500 μM) of MP-added medium to be cultured for 46 h, observed and recorded under an inverted microscope, and classified by Image J software (NIH, version number: 1.8.0 Official release, National Institutes of Health, Bethesda, MD, USA). To comprehend the specific evaluation system, refer to the results of this study.

### 2.3. Determination of ROS Levels

To measure the content of ROS in porcine oocytes, an ROS assay kit was applied [H2DCFDA (H2-DCF, DCF), ThermoFisher (Waltham, MA, USA). After 44–46 h of incubation, 10–20 oocytes from each treatment group were incubated with 10 μM H2DCFDA (diluted in PBS-PVA) for 30 min at 38.5 °C. After three washes with PBS, the samples were mounted on slides and imaged under an inverted fluorescence microscope (Ti2, Nikon, Tokyo, Japan). The fluorescence intensity of each sample was measured using the same scanning settings. Fluorescent pixel intensities were analyzed using Image J software (NIH, Bethesda, MD, USA). The excitation wavelengths used for FITC were 465–495 nm and emission wavelengths used for FITC were 512–558 nm.

### 2.4. Determination of GSH Levels

To analyze GSH levels in porcine oocytes, 10 µM 2′,7′-dichlorodihydrofluorescein diacetate (DCFH, Beyotime, Shanghai, China) was used. After 44–46 h of incubation, 10–20 detached oocytes from each treatment group were incubated with 10 μM H2DCFDA (diluted in PBS-PVA) for 30 min at 38.5 °C. After three washes with PBS, the samples were mounted on slides and imaged under an inverted fluorescence microscope (Ti2, Nikon). The fluorescence intensity of each sample was measured using the same scanning settings. Fluorescent pixel intensities were analyzed using Image J software (NIH). The excitation wavelengths used for DAPI were 362–396 nm and emission wavelengths used for DAPI were 432–482 nm.

### 2.5. Measurement of Mitochondrial Distribution

Here, 10 µM Mito-Tracker Red CMXRos (DCFH, Beyotime) was applied to assess the distribution and number of mitochondria in porcine oocytes. After 44–46 h of incubation, 10–20 detached oocytes from each treatment group were incubated with 10 μM Mito-Tracker Red (IVM dilution) for 30 min at 38.5 °C. After three washes with PBS, the samples were mounted on slides and imaged under an inverted fluorescence microscope (Ti2, Nikon) with the same scanning settings to measure the fluorescence intensity of each sample. Fluorescent pixel intensities were analyzed using Image J software (NIH, 1.8.0). The excitation wavelengths used for Tx Red were 540–580 nm and emission wavelengths used for Tx Red were 600–660 nm.

### 2.6. Determination of ATP Content

To analyze porcine oocyte ATP levels, after 44–46 h of incubation, 10–20 oocytes from each treatment group’s denuded oocytes were incubated with 100 μM ATP detection reagent for 30 min at 38.5 °C. After three washes with PBS, the samples were mounted on slides and imaged under an inverted fluorescence microscope (Ti2, Nikon). The fluorescence intensity of each sample was measured using the same scanning settings. Fluorescent pixel intensities were analyzed using Image J software (NIH). The excitation wavelengths used for FITC were 465–495 nm and emission wavelengths used for FITC were 512–558 nm.

### 2.7. Immunofluorescence

After the oocyte complexes were cultured for 44–46 h, the oocytes were denuded oocytes and washed five times with PBS-PVA. Subsequently, 3.7% paraformaldehyde was added and stored at 25 °C for 30 min. The oocytes were permeabilized with 0.3% Triton X-100 for 30 min at room temperature, and they were transferred to PBS-PVA containing 3% BSA and blocked for 1 h at 37 °C. The oocytes were incubated with the rabbit anti-LC3B antibody (#ab48394, Abcam, Cambridge, UK, 1:2000), rabbit anti-Nrf2 antibody (#3177, Cell Signaling Technology, Boston, MA, USA, 1:300), rabbit anti-CASP3 antibody (210392, Abcam, Cambridge, UK, 1:100), and rabbit anti-PGC1α+β antibody (AB72230, Abcam, Cambridge, UK, 1:100) overnight at −4 °C. After five washes with PBS-PVA, the cells were incubated with goat anti-rabbit IgG antibodies (#ab150077, Abcam, Cambridge, UK, 1:1000) for 1 h at 37 °C in the dark. For nuclear labeling, oocytes were treated with 10 µg/mL Hoechst 33342 at 37 °C for 10 min. Finally, the oocytes were washed five times in PBS-PVA before being mounted on slides with an anti-fluorescence attenuation sealant. Images were captured using a fluorescence microscope, and green/red fluorescence intensity was analyzed using ImageJ software.

### 2.8. Reverse Transcription-Polymerase Chain Reaction (RT-PCR)

COCs were cultured in IVM medium (M199 medium containing 0.022 mg/mL sodium pyruvate, 10% porcine follicular fluid, 0.09 mg/mL L-cysteine, 1% penicillin-streptomycin, 10 IU/mL follicle-stimulating hormone, 20 ng/mL epidermal growth factor, and 10 IU/mL LH) for 44–46 h. Total RNA was isolated from the oviductal oocyte complex using the Dynabeads mRNA Direct Purification Kit (Invitrogen, Carlsbad, CA, USA). RNA was reverse-transcribed to cDNA using the SuperScript III First Strand cDNA Synthesis Kit (Invitrogen). Subsequently, cDNA was analyzed for gene expression using the KAPA SYBR FAST universal qPCR kit (KAPA Biosystems, Boston, MA, USA). The total sample volume of the qPCR reaction was 20 µL, containing 1 µL (10 pmol) of each gene-specific primer, 10 µL of KAPA SYBR FAST qPCR Master Mix (2×) Universal, 7 µL of deionized water, and 1 µL of cDNA samples. The qPCR reaction conditions were as follows: initial polymerase activation at 95 °C for 180 s, followed by 40 cycles of denaturation at 95 °C for 3 s, annealing at 60 °C for 30 s, and extension at 72 °C for 20 s. A final extension was conducted at 72 °C for 5 min. GAPDH transcript levels were used for normalization, and gene expression was quantified using the 2^−∆∆Ct^ method. Each cDNA sample was analyzed three times. The primers used for RT-qPCR are listed in Table 1.

### 2.9. Statistical Analyses

For each set of experimental results, independent experiments were conducted at least three times. The data are expressed as the mean ± SEM. All data normalization processing and analysis follow this rule: in each experiment, the average value of the control group is calculated first, and then the control group is normalized, that is, the value of each control group is divided by the average value of the control group to obtain a group of control group normalized values. Similarly, the value of each treatment group was divided by the average value of the control group to obtain a normalized value of the treatment group. Finally, the normalized value was analyzed using SPASS software (v26.0, IBM Corporation, Armonk, NY, USA). Statistical analysis used SPSS software, and the differences between groups were assessed using the *t*-test or one-way ANOVA. Results at *p* < 0.05 were considered statistically significant.

## 3. Results

### 3.1. MP Exposure Affects Porcine Oocyte Maturation

To investigate the potential toxic effects of MP on porcine oocytes, we initially evaluated the maturation of oocytes treated with different concentrations (5, 50, and 500 μM) of MP-added medium for 46 h. PBI extrusion was used as the primary indicator for evaluating porcine oocyte maturation. The degree of cumulus expansion was assessed as another indicator of the effect of MP on oocyte maturation.

**Figure 1:**
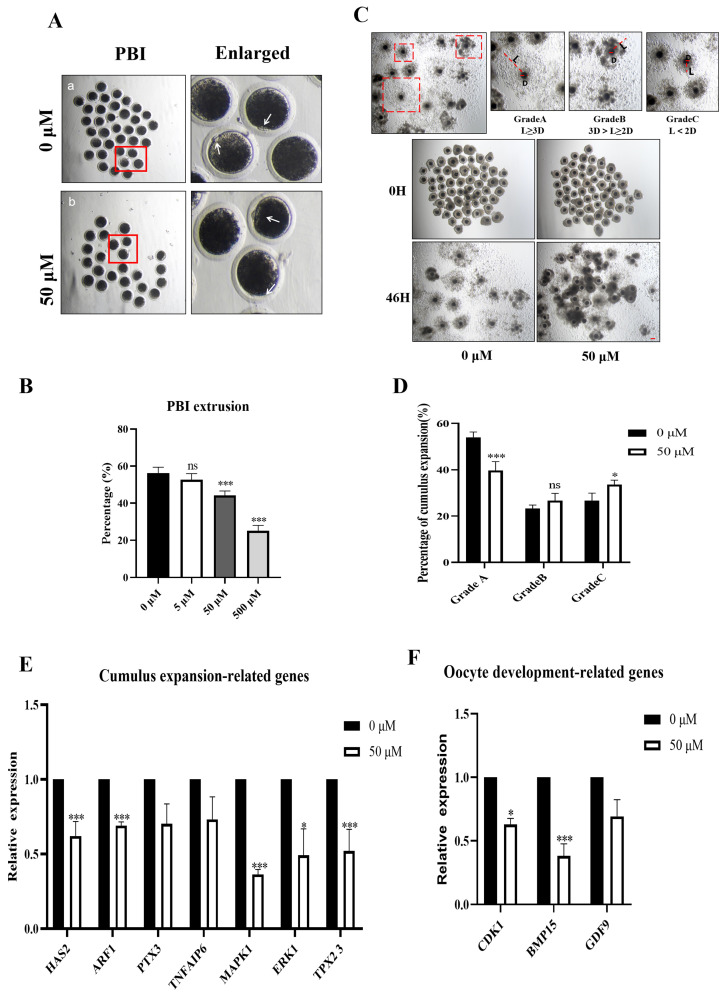
MP exposure affects porcine oocyte maturation. (**A**) Representative morphology of oocyte maturation after control and 50 μM MP exposure. Arrows indicate PBI. Scale bar = 100 μm. The white arrow marked in the enlarged image is a prominent polar body and is also used as a standard example of polar body discharge. The red box serves as the selection area for Posting large images. (**B**) Polar body extrusion rate in the control group and groups exposed to different concentrations (5, 50, and 500 μM) of MP. “ns” shows no difference, *** *p* < 0.001. (**C**) Representative images of three COCs with different degrees of expansion. A dashed red box of different expansion levels, with D indicating the oocyte radius and L indicating the cumulus expansion radius. Scale bar = 100 μm. (**D**) The date of COCs with different degrees of expansion. Difference is statistically significant. “***” indicates significance and “ns” shows no difference. * *p* < 0.05, *** *p* < 0.001. (**E**) Relative expansion of cumulus observed after control and 50 μM MP treatment. Grade A: fully expanded; Grade B: partially expanded; Grade C: poorly expanded. * *p* < 0.05. *** *p* < 0.001. (**F**) Expression of cumulus expansion-related genes detected in the control and 50 μM MP groups. * *p* < 0.05, *** *p* < 0.001. The error bars are representing the mean ± SEM. The *p*-values were calculated using Student’s *t*-test. The experiment was repeated 3 times for each group of data.

PBI extrusion was successful for the majority of oocytes in the control group, but failed for a significant percentage of oocytes after MP exposure. The results revealed no significant change in the polar body extrusion rate after 5 μM MP treatment (52.65 ± 1.92%, n = 228 oocytes, *p* < 0.05) compared with that in the control group (56.03 ± 2.05%, n = 230 oocytes). However, oocytes exposed to 50 and 500 μM MP exhibited significantly lower polar body extrusion rates in a dose-dependent manner. The polar body extrusion rate was decreased with increasing concentrations (50 μM: 44.17 ± 1.44%, n = 228 oocytes; 500 μM: 25.11 ± 1.69%, n = 224 oocytes; all *p* < 0.05; Figure 1A). Therefore, a concentration of 50 μM was selected for subsequent MP studies.

We examined the degree of the cumulus expansion of oocytes to further assess oocyte maturation following MP exposure. While the cumulus expansion was optimal in the control group, oocyte cumulus expansion was poor under MP exposure. The data were quantitatively analyzed by applying a method previously described by Xing et al. to assess the degree of oocyte mound expansion (Figure 1B) [25]. The degree of cumulus expansion was classified into grades A (fully expanded), B (partially expanded), and C (poorly expanded) by evaluating the relative expansion of cumulus in MP-exposed and control groups (L/D), oocyte diameter (D), and maximum diameter of cumulus diffusion (L); grade A expansion will be defined as the best degree of cumulus expansion (Figure 1C). The percentage of grade A cumulus expansion significantly decreased after 50 μM MP exposure (control(X): 54.25 ± 1.65%, n = 154 COCs vs. MP(Y): 38.00 ± 3.19%, n = 152; *p* < 0.05). The incidence of grade C cumulus expansion in the MP group was significantly higher than that in the control group (X: 26.00 ± 2.42%, n = 154 vs. Y: 37.5 ± 4.05%, n = 152; *p* < 0.05). However, no difference was observed in the percentage of grade B oval expansion between the control and MP groups (X: 24.17 ± 0.79%, n = 154 vs. Y: 26.67 ± 3.18%, n = 152; *p* < 0.05; Figure 1D).

To validate this finding and align the degree of cumulus expansion with the developmental outcome of oocytes, we then examined the expression levels of genes associated with cumulus expansion and oocyte development. The expression of genes related to cumulus expansion was significantly reduced in oocytes after 50 μM MP exposure compared with that in the controls (*HAS2*, control: 1.00 vs. MP: 0.62 ± 0.1, n = 218 COCs; *ARF1*, control: 1.00 vs. MP: 0.69 ± 0.03, n = 218 COCs; *MAPK1*, control: 1.00 vs. MP: 0.36 ± 0.03, n = 218 COCs; *ERK1*, control: 1.00 vs. MP: 0.33 ± 0.08, n = 218 COCs; *TPX2*, control: 1.00 vs. MP: 0.52 ± 0.1, n = 218 COCs; *P53*, control: 1.00 vs. MP: 0.56 ± 0.1, n = 218 COCs; all *p* < 0.05; Figure 1E). Although the difference in the expression levels of *PTX3* and *TNFAIP6* were not significant, their expression was decreased compared with that in controls. The expression of oocyte development-associated genes was significantly reduced after 50 μM MP exposure compared with that in the controls (*CDK1*, control: 1.00 vs. MP: 0.63 ± 0.04, n = 218 COCs; *BMP15*, control: 1.00 vs. MP: 0.37 ± 0.1, n = 218 COCs; all *p* < 0.05; Figure 1F). 

### 3.2. MP Exposure Affects Porcine Oocyte Redox Balance

**Figure 2:**
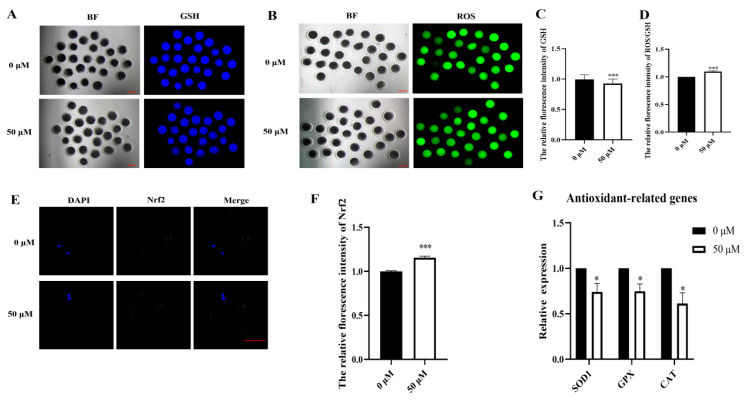
MP exposure affects redox homeostasis in porcine oocytes. (**A**) Representative images of reactive oxygen species (ROS) levels in the control and 50 μM MP-exposed oocytes. Scale bar = 100 μm. (**B**) Representative images of glutathione (GSH) levels after 50 μM MP exposure. (**C**) Relative fluorescence intensity of GSH analyzed in the control and 50 μM MP-exposed oocytes of date. *** *p* < 0.001. (**D**) Relative fluorescence intensity of ROS/GSH analyzed in the control and 50 μM MP-exposed oocytes. *** *p* < 0.001. (**E**) Representative images of nuclear factor erythroid 2-related factor 2 (Nrf2) levels analyzed in the control and 50 μM MP-exposed oocytes. (**F**) Relative fluorescence intensity of Nrf2 in the control and 50 μM MP-exposed oocytes. *** *p* < 0.001. (**G**) Relative expression of antioxidant-related genes in the control and 50 μM MP-exposed oocytes. * *p* < 0.05. The average fluorescence intensity of each oocyte was statistically analyzed. The error bars are representing the mean ± SEM. The *p*-values were calculated using Student’s *t*-test. The experiment was repeated 3 times for each group of data.

To investigate the influence of MP on oocyte redox balance, the levels of ROS and GSH in oocytes were examined, followed by an analysis of the GSH to ROS ratio. Although no significant change was observed in ROS fluorescence intensity in MP-exposed oocytes compared with that in the control, GSH fluorescence intensity exhibited a significant decrease (Figure 2A,B). This result was validated through fluorescence intensity analysis (control: 1.00, n = 40 oocytes vs. MP: 0.93 ± 0.01, n = 38 oocytes, *p* < 0.05; Figure 2C). Moreover, statistical analysis of the GSH to ROS ratio revealed a significant decrease (control: 1.00, n = 40 oocytes vs. MP: 1.10 ± 0.09, n = 38 oocytes, *p* < 0.05; Figure 2D), indicating a redox imbalance. This redox imbalance induces oxidative stress, prompting an evaluation of the expression of antioxidant-related genes and proteins. Our findings showed a significant decrease in the expression of antioxidant-related genes (*SOD1*, control: 1.00 vs. MP: 0.74 ± 0.09, n = 218 COCs; *GPX*, control: 1.00 vs. MP: 0.75 ± 0.08, n = 218 COCs; *CAT*, control: 1.00 vs. MP. 0.61 ± 0.1, n = 218 COCs; all *p* < 0.05; Figure 2E). The expression of the oxidative stress-related protein Nrf2 was significantly increased (control: 1.00, n = 40 oocytes vs. MP: 1.16 ± 0.02, n = 34 oocytes, *p* < 0.05; Figure 2F,G).

### 3.3. MP Exposure Affects Mitochondrial Function and Distribution in Porcine Oocytes

**Figure 3:**
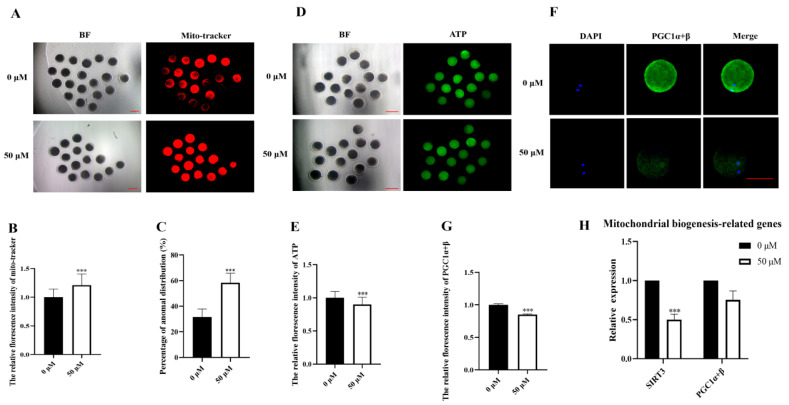
MP exposure affects mitochondrial function in porcine oocytes. (**A**) Representative images of mitochondria from the control and 50 μM MP-exposed oocytes. Scale bar = 100 μm. (**B**) Relative fluorescence intensity of mitochondria after 50 μM MP exposure. *** *p* < 0.001. (**C**) Mitochondrial abnormal distribution rate. *** *p* < 0.001. (**D**) Representative images of ATP content in the control and 50 μM MP-exposed oocytes of date. Scale bar = 100 μm. (**E**) Relative fluorescence intensity of ATP after 50 μM MP exposure. *** *p* < 0.001. (**F**) Representative images of Peroxisome proliferator-activated receptor gamma coactivator-1 alpha (PGC1α+β) content in the control and 50 μM MP-exposed oocytes. Scale bar = 100 μm. (**G**) Relative fluorescence intensity of PGC1α+β after 50 μM MP exposure. *** *p* < 0.001. (**H**) Relative expression of mitochondrial function-related genes in the control and 50 μM MP-exposed oocytes. *** *p* < 0.001. The average fluorescence intensity of each oocyte was statistically analyzed. The error bars are representing the mean ± SEM. The *p*-values were calculated using Student’s *t*-test. The experiment was repeated 3 times for each group of data.

Many studies have reported that the function of mitochondria affects the maturation of oocytes. Gene regulation can affect the normal function of the lineal body, such as the gene *SIRT3* and PGC1α+β protein that function normally in mitochondria. Mitochondrial dysfunction affects ATP production. It then affects the normal life activities of cells. To investigate the influence of mitochondria on porcine oocyte maturation, we initially examined the distribution of mitochondria. The majority of the mitochondria in the control group were distributed in the cortical layer, whereas most of the mitochondria in MP-exposed oocytes were located in the cytoplasm. The fluorescence intensity was significantly increased (Figure 3A). This observation was validated through fluorescence intensity analysis (control: 1.00, n = 47 oocytes vs. MP: 10.92 ± 0.02, n = 43 oocytes, *p* < 0.05; Figure 3B). Mitochondria primarily function to produce ATP for normal cellular activities. Impaired mitochondrial function in porcine oocytes results from sirtuin 3 (SIRT3) inhibition, leading to the disruption of mitochondrial membrane potential and reduced ATP levels [26]. Therefore, ATP content and SIRT3 expression levels were examined to assess mitochondrial function. The results revealed a significant decrease in ATP content in MP-exposed oocytes compared with that in controls (control: 1.00, n = 47 oocytes vs. MP: 0.92 ± 0.02, n = 43 oocytes, *p* < 0.05; Figure 3C,D). Additionally, SIRT3 expression was significantly decreased (control: 1.00 vs. MP: 0.50 ± 0.09, n = 218 COCs, *p* < 0.05; Figure 3E). PGC1α+β mediates mitochondrial function and biogenesis to maintain oocyte quality and fertility [27]. Therefore, the content of the PGC1α+β protein and the expression of genes associated with mitochondrial biogenesis were examined. The results indicated a significant decrease in PGC1α+β protein content, and this finding was corroborated through fluorescence intensity analysis (control: 1.00, n = 63 oocytes vs. MP: 0.85 ± 0.01, n = 63 oocytes, *p* < 0.05; Figure 3F). Although the decrease in the expression of genes related to mitochondrial biogenesis was not statistically significant, it was decreased compared with that in the control group.

### 3.4. MP Exposure Affects Autophagy and Apoptosis Levels in Porcine Oocytes

**Figure 4:**
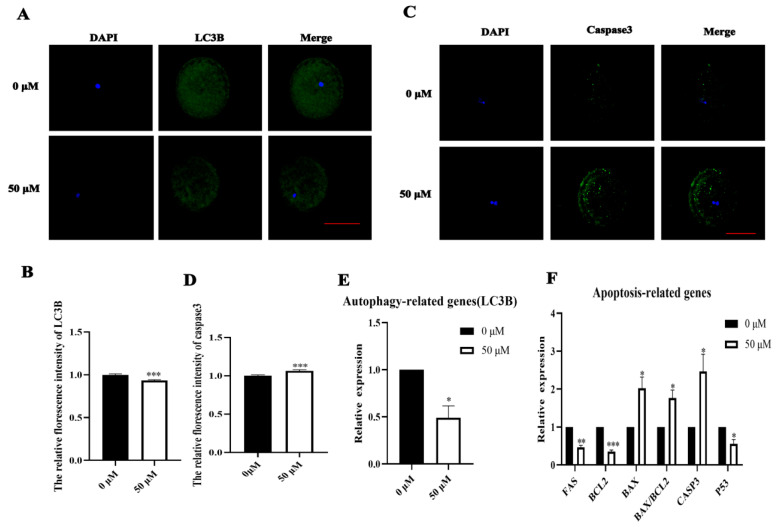
MP exposure affects autophagy and apoptosis levels in porcine oocytes. (**A**) Representative images of microtubule-associated protein 1 light chain 3 beta (LC3B) from the control and 50 μM MP-exposed oocytes. Scale bar = 100 μm. (**B**) Relative fluorescence intensity of microtubule-associated protein 1 light chain 3 beta (LC3B) after 50 μM MP exposure of date. *** *p* < 0.001 (**C**) Representative images of caspase3 levels in the control and 50 μM MP-exposed oocytes. Scale bar = 100 μm. (**D**) The relative florescence intensity of cysteine-requiring aspartate protease 3 (Caspase3) in the control and 50 μM MP-exposed oocytes of date. *** *p* < 0.001. (**E**) RT-PCR detection of autophagy-related gene expression in the control and 50 μM MP-exposed oocytes. * *p* < 0.05. (**F**) RT-PCR detection of apoptosis-related gene expression in the control and 50 μM MP-exposed oocytes. * *p* < 0.05, ** *p* < 0.01, *** *p* < 0.001. The average fluorescence intensity of each oocyte was statistically analyzed. The error bars are representing the mean ± SEM. The *p*-values were calculated using Student’s *t*-test. The experiment was repeated 3 times for each group of data.

During the developmental process of autophagy cells, a critical stage involves redox imbalance, which leads to increased apoptosis levels. Therefore, we examined the levels of autophagy-related protein LC3B and apoptosis-related protein caspase3, followed by an assessment of the expression levels of related genes. The study results indicated that the level of autophagy protein LC3B was significantly decreased (control: 1.00, n = 45 oocytes vs. MP: 0.93 ± 0.01, n = 41 oocytes, *p* < 0.05; Figure 4A,B). The level of apoptosis protein caspase3 was significantly increased (control: 1.00, n = 40 oocytes vs. MP: 1.06 ± 0.02, n = 40 oocytes, *p* < 0.05; Figure 4C,D). These findings were validated through fluorescence intensity analysis. Concurrently, the expression of apoptotic genes was significantly elevated (*FAS*, control: 1.00 vs. MP: 0.47 ± 0.05, n = 218 COCs; *CASP3*, control: 1.00 vs. MP: 2.46 ± 0.46, n = 218 COCs; *BAX*/*BCL2*, control: 1.00 vs. MP: 1.77 ± 0.21, n = 218 COCs; all *p* < 0.05; Figure 4E). The expression of autophagy genes was significantly decreased (*LC3B*, control: 1.00 vs. MP:0.49 ± 0.10, n = 218 COCs; *p* < 0.05; Figure 4F).

## 4. Discussion

In recent years, there has been a growing concern about the health risks associated with parabens due to their widespread use and potential endocrine-disrupting activities. Among them, MP is widely used in various aspects of life, yet studies on its reproductive toxicity remain scarce. In this study, we used porcine oocytes to explore the mechanism by which MP exposure during oocyte maturation affects development. The findings revealed that MP exposure hindered the developmental process of oocytes through redox imbalance and mitochondrial dysfunction.

During IVM, cumulus expansion and polar body extrusion are considered two key indicators of porcine oocyte maturation [28]. Additionally, the degree of cumulus expansion is related to the developmental quality of oocytes [29]. Therefore, these two indicators were examined in the present study. Embryonic development is susceptible to imperfections introduced during the process of oocyte maturation. Exposure of oocytes to inappropriate hormones inducing oocyte maturation in vitro in a culture system disrupts molecular reprogramming of oocytes and reduces subsequent developmental competence [30]. MP possesses estrogenic properties, and a positive correlation exists between MP and deleterious oxidative stress effects, cell proliferation, and cell viability [31]. Therefore, in the present study, the appropriate concentration of MP for investigation was selected through a series of concentration gradients. Oocytes exposed to 50 and 500 μM MP exhibited significantly lower polar body extrusion rates, indicating severe impairment of oocyte maturation. Studies on MP in oocytes are limited, but the significant impact of MP toxicity on oocyte maturation underscores the need for further investigation into its mechanism. Our study findings revealed that the degree of oocyte expansion was affected by MP, as evidenced by the decreased expression of oocyte expansion-related genes *ARF1*, *HAS2*, *MAPK1*, *ERK1*, *TPX2*, *PTX3*, and *TNFAIP6*. Although the difference in *GDF9* expression was not statistically significant, it was decreased compared with that in controls. Collectively, these results suggest that MP exposure may disrupt porcine oocyte maturation, warranting further investigation into its mechanism of toxicity during this process.

The overall redox balance of the oocyte complex is crucial for oocyte maturation and development. Reduced GSH levels are crucial for oocyte physiology, as they protect gametes from toxic ROS activity and are associated with porcine oocyte maturation [32]. Therefore, we examined the levels of ROS and GSH. The present study revealed an imbalance between ROS and GSH levels, indicative of an imbalance between oxidative and antioxidant capacities, signifying the occurrence of oxidative stress. Similar studies have reported that an altered GSH/ROS ratio adversely affects oocyte maturation [33], which is consistent with our findings. To further validate the impact of oxidative stress on oocyte maturation, we evaluated the content of the Nrf2 protein and the expression of relevant oxidation-related genes. Our results demonstrated a significant increase in Nrf2 protein content and mRNA levels of the gene *Nrf2* in MP-exposed oocytes. Previous studies have indicated a similar increase in the expression of total Nrf2 protein and nuclear proteins in porcine oocytes when exposed to H_2_O_2_ and the toxicant tunicamycin [34]. Additionally, mRNA levels of antioxidant-related genes (*SOD1*, *SOD2*, *CAT*, and *GPX*) were significantly decreased, further suggesting the presence of oxidative stress in oocytes. Therefore, our findings from the assessment of ROS/GSH, Nrf2 levels, and expression of related antioxidant genes indicate that mitochondrial dysfunction may be a potential causative factor.

Mitochondrial dysfunction adversely affects the normal development of oocytes [35]. This dysfunction impairs cellular function by decreasing ATP output and increasing oxidative stress. Moreover, it may lead to poor oocyte quality and embryo development, ultimately affecting pregnancy outcomes [36]. Therefore, we elucidated the mechanism of mitochondrial dysfunction in porcine oocyte maturation after MP exposure by examining ATP levels, mitochondrial distribution, and PGC1α+β content, which is a key protein related to mitochondrial biogenesis and function. Our results demonstrated a significant decrease in ATP levels in oocytes under MP exposure. Similar studies have indicated that oocytes exposed to toxic environments have impaired ATP production due to abnormal mitochondrial function [37]. Furthermore, the results revealed significantly elevated mitochondrial levels but with significantly abnormal distribution. Previous studies have suggested that damaged cells increase the number of mitochondria in response to stress to resist injury [38]. Oocytes exposed to toxicants induce abnormal mitochondrial distribution and reduced ATP levels [39]. PGC1α+β proteins determine most of the mitochondrial functions and are closely associated with mitochondrial biogenesis [40]. Our results showed a significant decrease in PGC1α+β protein levels, aligning with previous study findings indicating a significant decrease in PGCAα+β protein levels in oocytes exposed to cadmium, which is an environmental pollutant and toxicant [41]. SIRT3 plays a key role in oocyte maturation and alleviates polycystic ovary syndrome by modulating the FOXO1/PGC-1α+β signaling pathway. Further examination of related genes revealed decreased levels of SIRT3 and PGC1α+β expression.

Oxidative stress can activate autophagy by degrading damaged organelles or localized cytoplasm to mitigate oxidative damage and maintain normal cellular physiological activities [42]. Our results indicated a significant decrease in the levels of autophagy-related proteins and the associated gene *LC3B*. Previously, exposure to the endocrine disruptor nonylphenol has been shown to cause autophagy dysfunction by decreasing the level of autophagy-associated LC3B in oocytes, thereby affecting protein degradation [43]. Similar studies have also linked the maturation of oocytes exposed to toxic substances with decreased expression levels of autophagy proteins and genes [44], aligning with our experimental findings. Mitochondrial dysfunction, apoptosis, and oxidative stress are interrelated events [45]. Our experimental results revealed a significant decrease in related apoptosis-inhibiting genes *Bcl2*, *FAS*, and *P53*, and the expression of the pro-apoptotic related protein, gene caspase3, and the *BAX*/*BCL2* ratio was significantly increased. Adverse effects of butylbenzyl phthalate, which exhibits estrogenic effects, have been reported to involve the disruption of mitochondrial function and oxidative stress-induced early apoptosis [46]. The reproductive system of female Wistar rats exposed to the neonicotinoid insecticide thiamethoxam has been shown to result in follicles containing degenerated oocytes. Moreover, a significant increase in levels of apoptotic markers and high rates of apoptotic cell death have been reported [47]. These findings are consistent with our experimental results. Thus, exposure of oocytes to MP leads to autophagy dysfunction and induces apoptosis.

## 5. Conclusions

**Figure 5:**
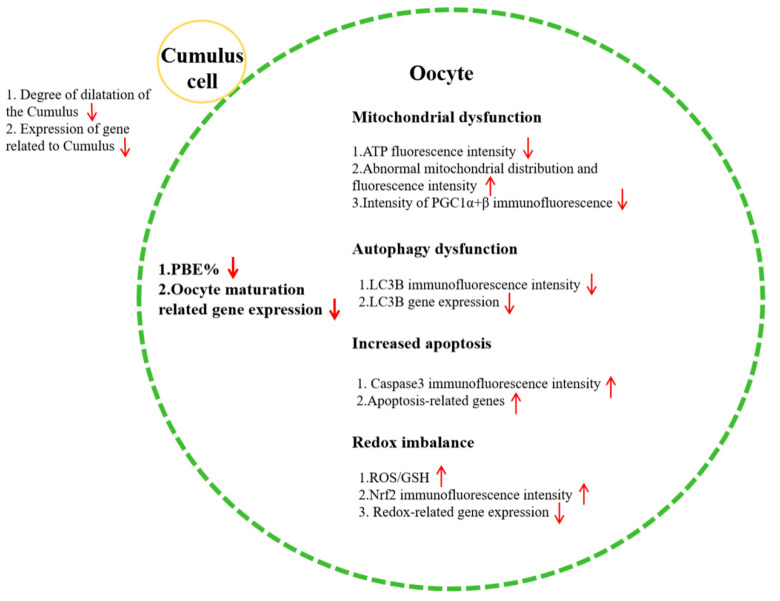
Schematic representation of the effects of MP exposure on porcine oocytes. The arrows indicate that the changes in cell related indicators in the 50μm treatment group. It is indicated increased when the arrow is up and it is indicated decreased when the arrow is down relative to the control group.

Exposure to MP affects mitochondrial function and distribution and the expression levels of biosynthesis-related genes and proteins in porcine oocytes. This exposure induces mitochondrial dysfunction and further disrupts the redox homeostasis of the oocytes, leading to exacerbated oxidative stress. Additionally, MP-exposed porcine oocytes display dysfunctional autophagy and increased apoptosis, thereby impeding their maturation (Figure 5).

## 6. Expect

In light of the findings from this study, the authors present a forward-looking perspective that emphasizes the importance of environmental protection and advocates for more rational approaches in the use of MP. This study also offers insights into monitoring such products; specifically, it highlights fluorescence detection methods as effective indicators for identifying pollutants. Previous research has indicated that cows are primarily exposed to MP through personal care products, suggesting that hair samples may serve as a suitable substrate for assessing MP levels in livestock [48]. 

Furthermore, alternative products should be considered due to the environmental release of the methyl paraben-containing items used in plastics, pharmaceuticals, and cosmetics. We can opt for reusable or environmentally friendly alternatives such as paper, cloth, or linen-based products. Lastly, it is advisable to select preservatives that are harmless upon degradation—examples include sodium lactate and lactostreptococcin. The authors also express hope that existing atmospheric microplastics and endocrine disruptors like MP can be effectively managed through various means such as capture technologies or biodegradation processes.

## Figures and Tables

**Table 1:** Primer sequences for RT-qPCR.

Genes	Forward Primers (5′ to 3′)	Reverse Primers (5′ to 3′)
*GAPDH*	GTCGGTTGTGGATCTGACCT	TTGACGAAGTGGTCGTTGAG
*HAS2*	AACGAACCGAGTGCTGAGTCTG	ACTTGCTCCAACGGGTCTGC
*ARF1*	AGCCGAGATCACCGACAAGC	CAGCCAGTCCAGTCCCTCATAG
*PTX3*	TGTGTGGGTGGTGGCTTTGATG	TGGGGCTGAATCTCTGTGACTCC
*TNFAIP6*	AGGCGAAAGCGGTGTGTGAATAC	ACCCAGCAGCACAGACATGAAATC
*GDF9*	CAGTCAGCTGAAGTGGGACA	TGGATGATGTTCTGCACCAT
*TPX2*	CTGAGGAGCAAGAATTGGAGAAGAG	TTTGGTTACCTGGGTCACTGATTTC
*ERK1*	CCTCCAACCTGCTCATCAACAC	ACATATTCCGTCAAGAAGCCAGTG
*MAPK1*	GGCTGTTCCCAAATGCTGACTC	CCTGCTCTACTTCAATCCTCTTGTG
*PGC1*α+β	AGGGAGAGGCAGAGGCAGAAG	TGTCCGTGTTGTGTCAGGTCTG
*SIRT3*	GTTCATCTGTTGCTGCCCTGAG	CTTCTTACTGTTGCCTCCACTTCC
*CAT*	AGCCAGTGACCAGATGAAGCATTG	ATGTCGTGTGTGACCTCAAAGTAGC
*GPX*	CTGGTCGTGCTCGGCTTCC	GCCTGGTCGGACGTACTTGAG
*SOD1*	CTCTCGGGAGACCATTCCATCATTG	TCCACCTCTGCCCAAGTCATCTG
*CASP3*	CGTGCTTCTAAGCCATGGTG	GTCCCACTGTCCGTCTCAAT
*BAX*	TGCCTCAGGATGCATCTACC	AAGTAGAAAAGCGCGACCAC
*BCL2*	AGGGCATTCAGTGACCTGAC	CGATCCGACTCACCAATACC
*FAS*	CACACCAACCAGCAACACCAAATG	AGGTACGGGAATGAGGATCAGGAG
*CDK1*	GGGCACTCCCAATAATGAAGT	GTTCTTGATACAACGTGTGGGAA
*BMP15*	CCTCCATCCTTTCCAAGTCA	GTGTAGTACCCGAGGGCAGA
*P53*	AATCCAGATGACGCCTCCAGAG	AGAAGGGACAAAGGACGACAGG
*LC3B*	GTGTAGTACCCGAGGGCAGA	TTTGGTAGGATGCTGCTCTCG

## Data Availability

The data presented in this study are available upon request from the corresponding author.

## References

[1] Guo Y., Kannan K. (2013). A survey of phthalates and parabens in personal care products from the United States and its implications for human exposure. Environ. Sci. Technol..

[2] Andersen M.H.G., Zuri G., Knudsen L.E., Mathiesen L. (2021). Placental transport of parabens studied using an ex-vivo human perfusion model. Placenta.

[3] Liang J., Liu Q.S., Ren Z., Min K., Yang X., Hao F., Zhang Q., Liu Q., Zhou Q., Jiang G. (2023). Studying paraben-induced estrogen receptor- and steroid hormone-related endocrine disruption effects via multi-level approaches. Sci. Total Environ..

[4] Rojo M., Ball A.L., Penrose M.T., Weir S.M., LeBaron H., Terasaki M., Cobb G.P., Lavado R. (2024). Accumulation of Parabens, Their Metabolites, and Halogenated Byproducts in Migratory Birds of Prey: A Comparative Study in Texas and North Carolina, USA. Environ. Toxicol. Chem..

[5] Nielsen N.J., Christensen P., Poulsen K.G., Christensen J.H. (2023). Investigation of micropollutants in household waste fractions processed by anaerobic digestion: Target analysis, suspect- and non-target screening. Environ. Sci. Pollut. Res. Int..

[6] Dasmahapatra A.K., Chatterjee J., Tchounwou P.B. (2024). A systematic review of the toxic potential of parabens in fish. Front. Toxicol..

[7] Soni M.G., Taylor S.L., Greenberg N.A., Burdock G.A. (2002). Evaluation of the health aspects of methyl paraben: A review of the published literature. Food Chem. Toxicol. Int. J. Publ. Br. Ind. Biol. Res. Assoc..

[8] Liang J., Yang X., Liu Q.S., Sun Z., Ren Z., Wang X., Zhang Q., Ren X., Liu X., Zhou Q. (2022). Assessment of Thyroid Endocrine Disruption Effects of Parabens Using In Vivo, In Vitro, and In Silico Approaches. Environ. Sci. Technol..

[9] Martins F.C., Oliveira M.M., Gaivão I., A. Videira R., Peixoto F. (2024). The administration of methyl and butyl parabens interferes with the enzymatic antioxidant system and induces genotoxicity in rat testis: Possible relation to male infertility. Drug Chem. Toxicol..

[10] Liu Y., Tao W., Wu S., Zhang Y., Nie H., Hou Z., Zhang J., Yang Z., Chen Z.J., Wang J. (2024). Maternal mRNA deadenylation is defective in in vitro matured mouse and human oocytes. Nat. Commun..

[11] Byskov A.G., Andersen C.Y., Leonardsen L. (2002). Role of meiosis activating sterols, MAS, in induced oocyte maturation. Mol. Cell. Endocrinol..

[12] Sharma A., Tiwari M., Gupta A., Pandey A.N., Yadav P.K., Chaube S.K. (2018). Journey of oocyte from metaphase-I to metaphase-II stage in mammals. J. Cell. Physiol..

[13] Jaffe L.A., Egbert J.R. (2017). Regulation of Mammalian Oocyte Meiosis by Intercellular Communication Within the Ovarian Follicle. Annu. Rev. Physiol..

[14] Hu W. (2009). The role of p53 gene family in reproduction. Cold Spring Harb. Perspect. Biol..

[15] Kim J.W., Park H.J., Yang S.G., Kim M.J., Kim I.S., Jegal H.G., Wee G., Yang H.Y., Park J.J., Choo Y.K. (2020). Exogenous Ganglioside GT1b Enhances Porcine Oocyte Maturation, Including the Cumulus Cell Expansion and Activation of EGFR and ERK1/2 Signaling. Reprod. Sci..

[16] Park H.J., Kim B., Koo D.B., Lee D.S. (2021). Peroxiredoxin 1 Controls Ovulation and Ovulated Cumulus-Oocyte Complex Activity through TLR4-Derived ERK1/2 Signaling in Mice. Int. J. Mol. Sci..

[17] Yokoo M., Kimura N., Sato E. (2010). Induction of oocyte maturation by hyaluronan-CD44 interaction in pigs. J. Reprod. Dev..

[18] Amaral S., S Tavares R., Baptista M., Sousa M.I., Silva A., Escada-Rebelo S., Paiva C.P., Ramalho-Santos J. (2016). Mitochondrial Functionality and Chemical Compound Action on Sperm Function. Curr. Med. Chem..

[19] Malott K.F., Luderer U. (2021). Toxicant effects on mammalian oocyte mitochondria. Biol. Reprod..

[20] Kirillova A., Smitz J.E.J., Sukhikh G.T., Mazunin I. (2021). The Role of Mitochondria in Oocyte Maturation. Cells.

[21] Tatemoto H., Sakurai N., Muto N. (2000). Protection of porcine oocytes against apoptotic cell death caused by oxidative stress during In vitro maturation: Role of cumulus cells. Biol. Reprod..

[22] Zhu Q., Ding D., Yang H., Zou W., Yang D., Wang K., Zhang C., Chen B., Ji D., Hao Y. (2022). Melatonin Protects Mitochondrial Function and Inhibits Oxidative Damage against the Decline of Human Oocytes Development Caused by Prolonged Cryopreservation. Cells.

[23] Fan L., Guan F., Ma Y., Zhang Y., Li L., Sun Y., Cao C., Du H., He M. (2022). N-Acetylcysteine improves oocyte quality through modulating the Nrf2 signaling pathway to ameliorate oxidative stress caused by repeated controlled ovarian hyperstimulation. Reprod. Fertil. Dev..

[24] Zhan C., Cao X., Zhang T., Guo J., Xu G., Wang H., Yang W., Yang L., Che D., Lu W. (2022). Melatonin protects porcine oocyte from copper exposure potentially by reducing oxidative stress potentially through the Nrf2 pathway. Theriogenology.

[25] Xing C., Chen S., Wang Y., Pan Z., Zou Y., Sun S., Ren Z., Zhang Y. (2022). Glyphosate exposure deteriorates oocyte meiotic maturation via induction of organelle dysfunctions in pigs. J. Anim. Sci. Biotechnol..

[26] Jiao L., Hu C.X., Zhang Y., Zhang Y.X., Cai W.W., Pan W.L., Sun S.C., Zhang Y. (2023). SIRT3 Regulates Levels of Deacetylated SOD2 to Prevent Oxidative Stress and Mitochondrial Dysfunction During Oocyte Maturation in Pigs. Microsc. Microanal. Off. J. Microsc. Soc. Am. Microbeam Anal. Soc. Microsc. Soc. Can..

[27] Wang R.S., Chang H.Y., Kao S.H., Kao C.H., Wu Y.C., Yeh S., Tzeng C.R., Chang C. (2015). Abnormal mitochondrial function and impaired granulosa cell differentiation in androgen receptor knockout mice. Int. J. Mol. Sci..

[28] Mesbah F., Kafi M., Nili H. (2016). Cumulus cell expansion and first polar body extrusion during in vitro oocyte maturation in relation to morphological and morphometric characteristics of the dromedary camel ovary. Reprod. Domest. Anim. = Zuchthyg..

[29] Appeltant R., Somfai T., Nakai M., Bodó S., Maes D., Kikuchi K., Van Soom A. (2015). Interactions between oocytes and cumulus cells during in vitro maturation of porcine cumulus-oocyte complexes in a chemically defined medium: Effect of denuded oocytes on cumulus expansion and oocyte maturation. Theriogenology.

[30] Moor R.M., Dai Y., Lee C., Fulka J. (1998). Oocyte maturation and embryonic failure. Hum. Reprod. Update.

[31] Elsehly W.M., Mourad G.M., Mehanna R.A., Kholief M.A., El-Nikhely N.A., Awaad A.K., Attia M.H. (2022). The potential implications of estrogenic and antioxidant-dependent activities of high doses of methyl paraben on MCF7 breast cancer cells. J. Biochem. Mol. Toxicol..

[32] Mateo-Otero Y., Yeste M., Damato A., Giaretta E. (2021). Cryopreservation and oxidative stress in porcine oocytes. Res. Vet. Sci..

[33] Leal G.R., Oliveira T.A., de Paula Guimarães M.P., Correia L.F.L., Vasconcelos E.M., Souza-Fabjan J.M.G. (2024). Lipid modulation during IVM increases the metabolism and improves the cryosurvival of cat oocytes. Theriogenology.

[34] Park H.J., Yang S.G., Koo D.B. (2022). SESN2/NRF2 signaling activates as a direct downstream regulator of the PERK pathway against endoplasmic reticulum stress to improve the in vitro maturation of porcine oocytes. Free Radic. Biol. Med..

[35] van der Reest J., Nardini Cecchino G., Haigis M.C., Kordowitzki P. (2021). Mitochondria: Their relevance during oocyte ageing. Ageing Res. Rev..

[36] Rodríguez-Varela C., Labarta E. (2020). Clinical Application of Antioxidants to Improve Human Oocyte Mitochondrial Function: A Review. Antioxidants.

[37] Hu L.L., Chen S., Shen M.Y., Huang Q.Y., Li H.G., Sun S.C., Wang J.L., Luo X.Q. (2023). Aflatoxin B1 impairs porcine oocyte quality via disturbing intracellular membrane system and ATP production. Ecotoxicol. Environ. Saf..

[38] Chen L., Huang J., Li X.C., Liu S.Y., Li Y.H., Wang Q., Yang J.J., Cao H.M., Hu Q.K., He L.J. (2019). High-glucose Induced Mitochondrial Dynamics Disorder of Spinal Cord Neurons in Diabetic Rats and its Effect on Mitochondrial Spatial Distribution. Spine.

[39] Wang Y., Pan Z.N., Xing C.H., Zhang H.L., Sun S.C. (2022). Nivalenol affects spindle formation and organelle functions during mouse oocyte maturation. Toxicol. Appl. Pharmacol..

[40] Scarpulla R.C. (2008). Transcriptional paradigms in mammalian mitochondrial biogenesis and function. Physiol. Rev..

[41] Idrees M., Kumar V., Khan A.M., Joo M.D., Uddin Z., Lee K.W., Kong I.K. (2022). Hesperetin activated SIRT1 neutralizes cadmium effects on the early bovine embryo development. Theriogenology.

[42] Liu X., Hussain R., Mehmood K., Tang Z., Zhang H., Li Y. (2022). Mitochondrial-Endoplasmic Reticulum Communication-Mediated Oxidative Stress and Autophagy. BioMed Res. Int..

[43] Hu L.L., Li H.G., Liao B.Y., Xu Y., Sun S.C., Wang J.L. (2022). Exposure to nonylphenol impairs oocyte quality via the induction of organelle defects in mice. Ecotoxicol. Environ. Saf..

[44] Zhang J.W., Xu D.Q., Feng X.Z. (2019). The toxic effects and possible mechanisms of glyphosate on mouse oocytes. Chemosphere.

[45] Modanloo M., Shokrzadeh M. (2019). Analyzing Mitochondrial Dysfunction, Oxidative Stress, and Apoptosis: Potential Role of L-carnitine. Iran. J. Kidney Dis..

[46] Jiang Y., Wang D., Zhang C., Jiao Y., Pu Y., Cheng R., Li C., Chen Y. (2023). Nicotinamide mononucleotide restores oxidative stress-related apoptosis of oocyte exposed to benzyl butyl phthalate in mice. Cell Prolif..

[47] El-Din M., Ghareeb A.E.E., El-Garawani I.M., El-Rahman H.A.A. (2023). Induction of apoptosis, oxidative stress, hormonal, and histological alterations in the reproductive system of thiamethoxam-exposed female rats. Environ. Sci. Pollut. Res. Int..

[48] Gonkowski S., Tzatzarakis M., Kadyralieva N., Vakonaki E., Lamprakis T. (2024). Exposure assessment of dairy cows to parabens using hair samples analysis. Sci. Rep..

